# A conceptual map of invasion biology: Integrating hypotheses into a consensus network

**DOI:** 10.1111/geb.13082

**Published:** 2020-03-25

**Authors:** Martin Enders, Frank Havemann, Florian Ruland, Maud Bernard‐Verdier, Jane A. Catford, Lorena Gómez‐Aparicio, Sylvia Haider, Tina Heger, Christoph Kueffer, Ingolf Kühn, Laura A. Meyerson, Camille Musseau, Ana Novoa, Anthony Ricciardi, Alban Sagouis, Conrad Schittko, David L. Strayer, Montserrat Vilà, Franz Essl, Philip E. Hulme, Mark van Kleunen, Sabrina Kumschick, Julie L. Lockwood, Abigail L. Mabey, Melodie A. McGeoch, Estíbaliz Palma, Petr Pyšek, Wolf‐Christian Saul, Florencia A. Yannelli, Jonathan M. Jeschke

**Affiliations:** ^1^ Department of Biology, Chemistry, Pharmacy Institute of Biology Freie Universität Berlin Berlin Germany; ^2^ Leibniz‐Institute of Freshwater Ecology and Inland Fisheries (IGB) Berlin Germany; ^3^ Berlin‐Brandenburg Institute of Advanced Biodiversity Research (BBIB) Berlin Germany; ^4^ Philosophische Fakultät Institut für Bibliotheks‐ und Informationswissenschaft Humboldt‐Universität zu Berlin Berlin Germany; ^5^ Department of Geography King’s College London London United Kingdom; ^6^ School of BioSciences The University of Melbourne Parkville Victoria Australia; ^7^ Biological Sciences University of Southampton Southampton United Kingdom; ^8^ Instituto de Recursos Naturales y Agrobiología de Sevilla (IRNAS), CSIC Seville Spain; ^9^ Martin Luther University Halle‐Wittenberg Institute of Biology/Geobotany and Botanical Garden Halle (Saale) Germany; ^10^ German Centre for Integrative Biodiversity Research (iDiv) Halle‐Jena‐Leipzig Leipzig Germany; ^11^ Biodiversity Research/Systematic Botany University of Potsdam Potsdam Germany; ^12^ Technical University of Munich Freising Germany; ^13^ Institute of Integrative Biology, Department of Environmental Systems Science ETH Zurich Zurich Switzerland; ^14^ Centre for Invasion Biology Department of Botany and Zoology Stellenbosch University Matieland South Africa; ^15^ Helmholtz Centre for Environmental Research – UFZ Department Community Ecology Halle (Saale) Germany; ^16^ The University of Rhode Island Department of Natural Resources Science Kingston Rhode Island; ^17^ Czech Academy of Sciences Institute of Botany Department of Invasion Ecology Průhonice Czech Republic; ^18^ Redpath Museum McGill University Montreal Quebec Canada; ^19^ Cary Institute of Ecosystem Studies Millbrook New York United States; ^20^ Graham Sustainability Institute University of Michigan Ann Arbor Michigan United States; ^21^ Estación Biológica de Doñana (EBD‐CSIC) Seville Spain; ^22^ Department of Plant Biology and Ecology University of Seville Seville Spain; ^23^ Department of Botany and Biodiversity Research University of Vienna Vienna Austria; ^24^ Bio‐Protection Research Centre Lincoln University Lincoln, Canterbury New Zealand; ^25^ Ecology, Department of Biology University of Konstanz Konstanz Germany; ^26^ Zhejiang Provincial Key Laboratory of Plant Evolutionary Ecology and Conservation Taizhou University Taizhou China; ^27^ South African National Biodiversity Institute Kirstenbosch National Botanical Gardens Claremont South Africa; ^28^ Ecology, Evolution and Natural Resources Rutgers University New Brunswick New Jersey; ^29^ Ocean and Earth Science National Oceanography Centre University of Southampton Southampton United Kingdom; ^30^ School of Biological Sciences Monash University Clayton Victoria Australia; ^31^ Department of Ecology Faculty of Science Charles University Prague Czech Republic; ^32^ Centre for Invasion Biology Department of Mathematical Sciences Stellenbosch University Matieland South Africa

**Keywords:** biological invasions, concepts, consensus map, Delphi method, invasion science, invasion theory, navigation tools, network analysis

## Abstract

**Background and aims:**

Since its emergence in the mid‐20th century, invasion biology has matured into a productive research field addressing questions of fundamental and applied importance. Not only has the number of empirical studies increased through time, but also has the number of competing, overlapping and, in some cases, contradictory hypotheses about biological invasions. To make these contradictions and redundancies explicit, and to gain insight into the field’s current theoretical structure, we developed and applied a Delphi approach to create a consensus network of 39 existing invasion hypotheses.

**Results:**

The resulting network was analysed with a link‐clustering algorithm that revealed five *concept clusters* (resource availability, biotic interaction, propagule, trait and Darwin’s clusters) representing complementary areas in the theory of invasion biology. The network also displays hypotheses that link two or more clusters, called *connecting hypotheses*, which are important in determining network structure. The network indicates hypotheses that are logically linked either positively (77 connections of support) or negatively (that is, they contradict each other; 6 connections).

**Significance:**

The network visually synthesizes how invasion biology’s predominant hypotheses are conceptually related to each other, and thus, reveals an emergent structure – a *conceptual map* – that can serve as a navigation tool for scholars, practitioners and students, both inside and outside of the field of invasion biology, and guide the development of a more coherent foundation of theory. Additionally, the outlined approach can be more widely applied to create a conceptual map for the larger fields of ecology and biogeography.

## 1 INTRODUCTION

The first author’s grandfather was a master electrician working for the city of Munich, Germany, whose daily work consisted of repairing streetlights and other electrical devices for public use. One of his most impressive skills was his ability to intimately recall the details of every place in his district. By combining his knowledge with that of co‐workers familiar with other districts, one could have created a complete map of the city that would allow anyone to confidently navigate its streets. In many ways, a research field is quite similar to a city where its major questions and hypotheses represent subunits comparable to city districts. Such subunits can be represented on a map, whether of a city or a research field, the latter allowing scientists inside and outside of the field to better orientate themselves and navigate their own research interests. Such a map would also be useful for students, teachers, policymakers and managers, as it would allow them to efficiently identify the elements of science most pertinent to their interests and goals.

Some previous conceptual maps of science take the form of networks, and cover multiple disciplines; that is, they chart science as a whole and show how different disciplines relate to each other (Börner, 2010, 2015). These maps usually do not focus on the theory of any one discipline, and thus, do not represent the myriads of hypotheses and concepts of each research field. Given that concepts and hypotheses form the backbone of scientific inquiry, we posit that it is useful to simultaneously create conceptual maps within research disciplines to visualize the relationships among key hypotheses (Jeschke, 2014). Conceptual maps identify the degree to which hypotheses are similar, competing or contradictory, and use this information to aggregate them into broader clusters.

Conceptual maps in the form of networks can be particularly useful for disciplines with many hypotheses, where even researchers within the field tend to be restricted to specific research silos and are thereby increasingly unaware of similar hypotheses in the field. An example of such a discipline is invasion biology. Since the emergence of the field with the publication of Charles Elton’s book *The ecology of invasions by animals and plants* in 1958 and sustained research programmes developed in the 1990s (Richardson & Pyšek, 2008), it has accumulated an impressive number of hypotheses and concepts (see Table 1 for references and descriptions of the hypotheses). A recent online survey indicated that many invasion biologists appear to be knowledgeable about hypotheses and concepts they are directly working with, but do not demonstrate a consistent understanding of the relationship among these and other concepts in the field (Enders, Hütt, & Jeschke, 2018).

**TABLE 1:** List of 39 common invasion hypotheses and how they were defined for this study [adapted from Catford et al. (2009) and Enders et al. (2018)]

Hypothesis		Description	Key reference(s)
ADP	Adaptation	The invasion success of non‐native species depends on the adaptation to the conditions in the exotic range before and/or after the introduction. Non‐native species that are related to native species are more successful in this adaptation	Duncan and Williams (2002)
BA	Biotic acceptance aka ‘the rich get richer’	Ecosystems tend to accommodate the establishment and coexistence of non‐native species despite the presence and abundance of native species	Stohlgren, Jarnevitch, and Chong (2006)
BID	Biotic indirect effects	Non‐native species benefit from different indirect effects triggered by native species	Callaway, Thelen, Rodriguez, and Holben (2004)
BR	Biotic resistance aka diversity‐invasibility hypothesis	An ecosystem with high biodiversity is more resistant against non‐native species than an ecosystem with lower biodiversity	Elton (1958); Levine and D'Antonio (1999)
CP	Colonization pressure	Colonization pressure is defined as the number of species introduced to a given location. As colonization pressure increases, the number of established or invasive non‐native species in that location is predicted to increase	Lockwood, Cassey, and Blackburn (2009)
DEM	Dynamic equilibrium model	The establishment of a non‐native species depends on natural fluctuations of the ecosystem, which influence the level of competition from local species	Huston (1979)
DN	Darwin’s naturalization	The invasion success of non‐native species is higher in areas that are poor in closely related species than in areas that are rich in closely related species	Daehler (2001); Darwin (1859)
DS	Disturbance	The invasion success of non‐native species is higher in highly disturbed than in relatively undisturbed ecosystems	Elton (1958); Hobbs and Huenneke (1992)
EIM	Ecological imbalance	Invasion patterns are a function of the evolutionary characteristics of both the recipient region and potential donor regions. Species from regions with highly diverse evolutionary lineages are more likely to become successful invaders in less diverse regions	Fridley and Sax (2014)
ENA	Ecological naivety aka evolutionary naivety aka eco‐evolutionary naivety	The impact of a non‐native species on biodiversity is influenced by the evolutionary experience of the invaded community. Thus, the largest impacts are caused by species (e.g., predators, herbivores, pathogens) invading systems where no phylogenetically or functionally similar species exist	Diamond and Case (1986); Ricciardi and Atkinson (2004)
EE	Enemy of my enemy aka accumulation‐of‐local‐pathogens hypothesis	Introduced enemies of a non‐native species are less harmful to the non‐native than to the native species	Eppinga et al. (2006)
EI	Enemy inversion	Introduced enemies of non‐native species are less harmful for them in the exotic than the native range, due to altered biotic and abiotic conditions	Colautti, Ricciardi, Grigorovich, and MacIsaac (2004)
EICA	Evolution of increased competitive ability	After having been released from natural enemies, non‐native species will allocate more energy in growth and/or reproduction (this re‐allocation is due to genetic changes), which makes them more competitive	Blossey and Nötzold (1995)
EN	Empty niche	The invasion success of non‐native species increases with the availability of empty niches in the exotic range	MacArthur (1970)
ER	Enemy release	The absence of enemies in the exotic range is a cause of invasion success	Keane and Crawley (2002)
ERD	Enemy reduction	The partial release of enemies in the exotic range is a cause of invasion success	Colautti et al. (2004)
EVH	Environmental heterogeneity	The invasion success of non‐native species is high if the exotic range has a highly heterogeneous environment	Melbourne et al. (2007)
GC	Global competition	A large number of different non‐native species is more successful than a small number	Colautti, Grigorovich, and MacIsaac (2006)
HC	Human commensalism	Species that live in close proximity to humans are more successful in invading new areas than other species	Jeschke and Strayer (2006)
HF	Habitat filtering	The invasion success of non‐native species in the new area is high if they are pre‐adapted to this area	Weiher and Keddy (1995)
IM	Invasional meltdown	The presence of non‐native species in an ecosystem facilitates invasion by additional species, increasing their likelihood of survival or ecological impact	Simberloff and Holle (1999)
IRA	Increased resource availability	The invasion success of non‐native species increases with the availability of resources	Sher and Hyatt (1999)
IS	Increased susceptibility	If a non‐native species has a lower genetic diversity than the native species, there will be a low probability that the non‐native species establishes itself	Colautti et al. (2004)
ISH	Island susceptibility hypothesis	Non‐native species are more likely to become established and have major ecological impacts on islands than on continents	Jeschke (2008)
IW	Ideal weed	The invasion success of a non‐native species depends on its specific traits (e.g., life‐history traits)	Baker (1965); Rejmánek and Richardson (1996)
LS	Limiting similarity	The invasion success of non‐native species is high if they strongly differ from native species, and low if they are similar to native species	MacArthur and Levins (1967)
MM	Missed mutualisms	In their exotic range, non‐native species suffer from missing mutualists	Mitchell et al. (2006)
NAS	New associations	New relationships between non‐native and native species can positively or negatively influence the establishment of the non‐native species	Colautti et al. (2006)
NW	Novel weapons	In the exotic range, non‐native species can have a competitive advantage against native species because they possess a novel weapon, that is, a trait that is new to the resident community of native species and, therefore, affects them negatively	Callaway and Ridenour (2004)
OW	Opportunity windows	The invasion success of non‐native species increases with the availability of empty niches in the exotic range, and the availability of these niches fluctuates spatio‐temporally	Johnstone (1986)
PH	Plasticity hypothesis	Invasive species are more phenotypically plastic than non‐invasive or native ones	Richards, Bossdorf, Muth, Gurevitch, and Pigliucci (2006)
PO	Polyploidy hypothesis	Polyploid organisms, particularly plants, are predicted to have an increased invasion success, since polyploidy can lead to higher fitness during the establishment phase and/or increased potential for subsequent adaptation	te Beest et al. (2012)
PP	Propagule pressure	A high propagule pressure (a composite measure consisting of the number of individuals introduced per introduction event and the frequency of introduction events) is a cause of invasion success	Lockwood, Cassey, and Blackburn (2005)
RER	Resource‐enemy release	The non‐native species is released from its natural enemies and can spend more energy in its reproduction, and invasion success increases with the availability of resources	Blumenthal (2006)
RI	Reckless invader aka ‘boom‐bust’	A population of a non‐native species that is highly successful shortly after its introduction can decline or disappear over time due to different reasons (such as competition with other introduced species or adaptation by native species)	Simberloff and Gibbons (2004)
SDH	Shifting defence hypothesis	After having been released from natural specialist enemies, non‐native species will allocate more energy to cheap (energy‐inexpensive) defences against generalist enemies and less energy to expensive defences against specialist enemies (this re‐allocation is due to genetic changes); the energy gained in this way will be invested in growth and/or reproduction, which makes the non‐native species more competitive	Doorduin and Vrieling (2011)
SG	Specialist‐generalist	Non‐native species are more successful in a new region if the local predators are specialists and local mutualists are generalists	Callaway et al. (2004)
SP	Sampling	A large number of different non‐native species is more likely to become invasive than a small number due to interspecific competition. Also, the species identity of the locals is more important than the richness in terms of the invasion of an area	Crawley, Brown, Heard, and Edwards (1999)
TEN	Tens rule	Approximately 10% of species successfully take consecutive steps of the invasion process	Williamson and Brown (1986)

Some invasion hypotheses are more popular in particular taxa or subfields of invasion biology than in others. For example, analysing over 1,000 studies concerning 10 invasion hypotheses, Jeschke and Heger (2018: table 17.2) found that four of these hypotheses are predominantly addressed by studies on non‐native animals. Of the studies addressing the island susceptibility hypothesis, 65% focused on non‐native vertebrates (see also Jeschke et al., 2012); of the studies addressing the limiting similarity hypothesis, only 3% focused on vertebrates and 94% on plants. Cross‐taxonomic studies are rare for most hypotheses, with invasional meltdown being a notable exception (Jeschke et al., 2012). This hypothesis has also been addressed by a substantial number of studies in aquatic habitats (37%), whereas other hypotheses have been predominantly investigated in terrestrial habitats: of the nine other hypotheses analysed by Jeschke and Heger (2018: table 17.3), the average proportion of terrestrial studies was 84%. The overall clear pattern is that different invasion hypotheses are investigated within different taxonomic groups and different habitats. In addition, the hypotheses represent different perspectives on biological invasions: some focus on ecosystem properties (e.g., empty niche hypothesis), others on interactions with humans (e.g., propagule pressure hypothesis), biotic interactions (e.g., enemy release hypothesis) or species traits (e.g., ideal weed hypothesis).

Given that many researchers and conservationists working in the various subfields of invasion biology no longer appear to have a good overview of the general discipline’s major theoretical ideas (as indicated by Enders et al., 2018), a network of concepts, representing a conceptual map of invasion biology, would provide much‐needed orientation and navigation. Because maps can take the form of networks, we use both terms in a similar way: *network* is the more technical term and better describes how the map is methodologically constructed, whereas the term *map* focuses on the purpose as a navigation tool.

Several approaches have previously been used to visualize a network of invasion hypotheses, although they have some limitations. These attempts build on past work that highlighted commonalities among invasion hypotheses but did not visualize them (Catford, Jansson, & Nilsson, 2009). First, Enders et al. (2018) created a network by asking researchers which hypotheses they knew best. This approach assumes that if many researchers state that they know a given pair of hypotheses very well, these hypotheses probably have something in common, and can thus be connected in a network. This is a ‘black box’ approach, as it is unclear why researchers often know a certain pair of hypotheses well and thus what a connection between hypotheses really means.

Second, Enders and Jeschke (2018) assessed the conceptual similarity of hypotheses by classifying which factors are highlighted as most important for the invasion success of non‐native species. The resulting table characterizing the hypotheses (based on Catford et al., 2009) was then used to create a network showing conceptual overlaps. A weakness of this approach is that the classification was based on the assessments of very few experts, namely the authors of Catford et al. (2009; *n* = 3) and Enders and Jeschke (2018; *n* = 2).

Finally, Enders, Havemann, and Jeschke (2019) applied a bibliometric approach to create a network of invasion hypotheses. In their network, two hypotheses are connected if key publications featuring these hypotheses are frequently cited together. Co‐citation analysis was recently also applied by Trujillo and Long (2018) who created a sequence of nested co‐citation networks (although these are not hypothesis networks). The application of co‐citation analysis for creating hypothesis networks has three main limitations: (a) a publication may be cited for reasons other than the hypothesis that it refers to; (b) it is not possible to discriminate among hypotheses that support one another and those that contradict one another; and (c) especially in large, complex fields, research areas that are logically connected are not always bibliographically connected (Swanson, 1986).

To overcome the limitations of these approaches, we present a novel consensus approach based on the Delphi method to create a network of invasion hypotheses that capitalizes on the expertise of a group of invasion biologists who work on different topics and various taxonomic groups and habitats. The approach can be generally applied to any research field; thus, invasion biology is used as a case example here. In a Delphi method, the opinions of a group of experts converge towards a consensus in several steps during which the experts revise their opinion based on an anonymized summary of all experts’ opinions (Häder & Häder, 2000). In the resulting consensus network, we identified hypothesis clusters by applying a state‐of‐the‐art link‐clustering algorithm.

## 2 METHODS

### 2.1 Consensus approach

**FIGURE 1:**
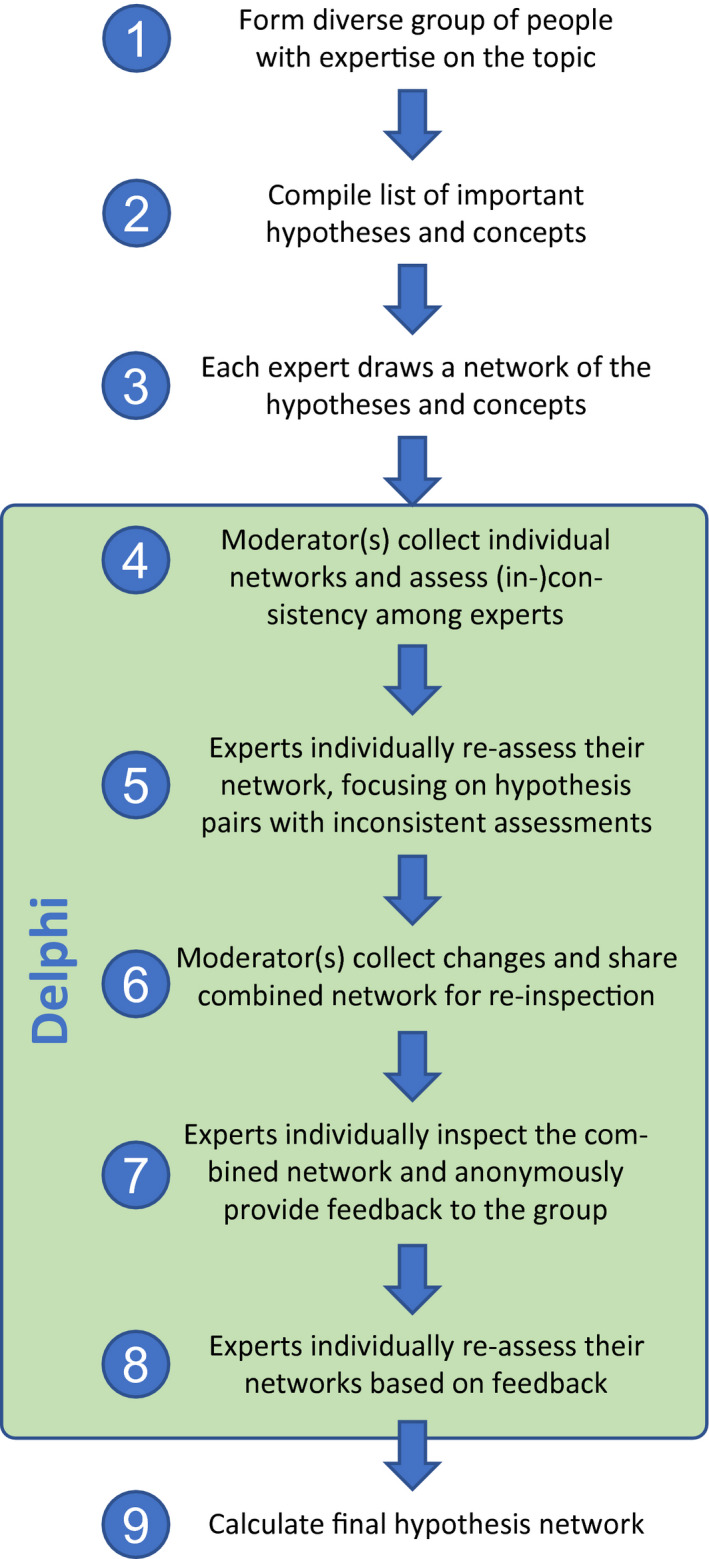
Description of the consecutive steps to create a consensus network of hypotheses and concepts. While we applied this approach for the field of invasion biology, it can be easily applied for other research fields as well [Colour figure can be viewed at wileyonlinelibrary.com]

Our approach to creating a consensus network of invasion hypotheses consists of nine steps (Steps 4 to 8 represent the Delphi approach; Figure 1). In Step 1, a group of 29 experts in invasion biology were assembled to ensure a breadth of experience, wide taxonomic knowledge and geographic scope. Given the high level of expertise needed for the task, of the 29 experts, 15 were senior scientists (52%), 10 postdocs or on a similar level (34%) and 4 were PhD students (14%). Gender representation was roughly equal with 14 male (48%) and 15 female (52%) group members. Of the 29 experts, 19 were based in Europe (66%), 4 in North America (14%), 3 in Africa (10%) and 3 in Australasia (10%). Eighteen of these 29 invasion biologists plus Frank Havemann, an expert on network analysis, met in Berlin on 12–13 February 2018. The European location of the meeting (and associated logistical constraints) resulted in the over‐representation of European researchers. Follow‐up communication with all participants was through e‐mail.

In Step 2, the moderators (ME, FR and JMJ) compiled a list of 39 hypotheses and concepts related to the invasion stages of introduction, establishment and spread, with reference to the respective original publication author/s and year (Table 1). This list, which expanded the 33 hypotheses listed by Enders et al. (2018) by 6 additional hypotheses considered to be influential by the experts, is one of the most extensive lists of invasion hypotheses compiled to date (together with Chabrerie et al., 2019).

In Step 3, we asked the experts to build their own version of the network. Each of the 29 experts was given the option of following one of two approaches: (a) to draw a network of the 39 hypotheses, with similar hypotheses connected by a black line, contradictory hypotheses connected by a red line, and other hypotheses (which are not logically linked) unconnected; or (b) to assess the similarity of hypotheses in a matrix by giving a value of 1 for a pair of similar hypotheses, a value of −1 for contradictory hypotheses, and 0 for hypotheses that are not logically linked, not even in a contradictory way. Hypothesis pairs could be left aside and indicated with ‘NA’ if an expert felt uncomfortable making a decision about the similarity of these hypotheses. However, this option was rarely chosen by the participants (0.53%). Each expert then individually sent their network or matrix to the moderators.

A key aspect of Step 3 is that researchers may have a different interpretation of the terms ‘similar’ and ‘contradictory’. We collectively agreed that both terms mean two hypotheses are logically linked; we call them ‘similar’ if they are positively linked, and ‘contradictory’ if they are negatively linked. Beyond this definition, participants were free to decide what a ‘logical link’ means. This freedom allowed us to capture the diverse backgrounds and perspectives of individuals in the group. Most participants evaluated a logical link primarily based on the ecological mechanisms described in the hypotheses (e.g., hypotheses are logically linked if they both consider a certain type of biotic interaction), whereas some respondents included the level of organization (genotype, individual, population, community) or the indirect effects of an invasion in their link evaluation. Others considered which hypotheses gave rise to, or were cited by, another hypothesis; or to which degree the knowledge of one hypothesis substantially informs our understanding of another (e.g., understanding the enemy release hypothesis can be seen as fundamental for understanding the enemy reduction hypothesis), especially if the outcomes of both hypotheses go in the same direction (e.g., lack of enemies increases invasion success).

In Step 4, the moderators received the individual assessments and calculated the percentage of respondents who indicated hypotheses that are logically linked either positively (+1, i.e., similar hypotheses) or negatively (−1, i.e., contradictory hypotheses). For this calculation, NA scores were excluded. For example, given the entries for a hypothesis pair are: 0, 0, 1, −1, 1, −1, NA, 0, 1, 1, NA, 1, the percentage of +1 or −1 values compared to zeros for this response set would be 7/10 = .7 (2 × NA, 3 × 0, 5 × 1, 2 × −1). We then determined the sign of the connection (positive or negative) based on the majority of individual entries. In the example before, there are five entries with +1 and two entries with −1, thus the overall sign of the connection is positive. The overall score for this hypothesis pair would thus be +.7. We never found that the numbers of negative and positive signs were the same; in such a case, we would have asked the experts to re‐assess the connection. The final action in Step 4 was to discriminate (a) hypothesis pairs for which most participants agreed that the hypotheses are either similar (overall value > .65), contradictory (< −.65) or not logically linked (value between −.35 and .35) from (b) hypothesis pairs for which the entries were inconclusive (value close to ±.5: between −.65 and −.35, or between .35 and .65). The value of ±.65 as a decision rule was set by the group.

In Step 5, all participants were asked to re‐inspect hypothesis pairs with inconclusive entries (that was the case for 52 hypothesis pairs) and to individually send their revised network or matrix to the moderators.

In Step 6, the moderators calculated an overall hypothesis network based on the links among hypotheses, using the R statistical environment version 3.1.0 (R Development Core Team, 2018) and packages ‘sna’ (Handcock, Hunter, Butts, Goodreau, & Morris, 2003), ‘reshape2’ (Wickham, 2007) and ‘igraph’ (Csardi & Nepusz, 2006) (see below for details) and shared it with all participants.

In Step 7, the participants inspected the overall network, and those who disagreed with any element explained their reason for this disagreement by sending an individual e‐mail to the moderators who then shared the collected and anonymized feedback with the group.

In Step 8, participants inspected their assessments again based on this feedback and sent their final network or matrix to the moderators if any changes were made. All individual networks are provided in Supporting Information Table S1.

In Step 9, the moderators calculated final values for the link between each pair of hypotheses (Supporting Information Table S1) and constructed the final hypothesis network.

### 2.2 Clustering approach

To reveal the inner structure of a network, it is helpful to group the nodes (in our case: the hypotheses) of the network into clusters. A common way of doing so is node clustering, for which various algorithms exist (Fortunato, 2010). We applied four established node‐clustering algorithms which, however, led to different network clusters (see Supporting Information Appendix S1). These inconsistencies were largely due to the fact that some hypotheses did not seem to be part of any single cluster, but were instead bridging clusters. We therefore decided to apply a link‐clustering method instead (Ahn, Bagrow, & Lehmann, 2010; Evans & Lambiotte, 2009), an approach that allows for nodes to be members of multiple clusters. Link clustering is thus more flexible than node clustering where each node can only be in one cluster (see Supporting Information Appendix S1 for details).

Clusters of links induce node communities whereby the membership grade of each node to the community induced by link cluster *L* is given by the proportion of its internal links $\frac{k_{i}^{\mathrm{in}}\left(L\right)}{k_{i}}$ (see below for details). Because we assumed that pairs of similar hypotheses identified in one region of the network are independent of hypothesis pairs in other regions, we chose a local approach to link clustering, where each link set *L* is evaluated independently from the rest of the network (Havemann, Gläser, & Heinz, 2017). Local link clustering allows for communities not only to overlap in boundary nodes, but also in inner nodes. One measure for evaluating link clusters is the escape probability of the link–node–link random walker. This random walker – introduced by Evans and Lambiotte (2009) – is the translation of the ordinary random walker into the world of link clustering. The walker starts from a link, goes randomly to one of its nodes, and then to one of the links of this node. If the escape probability is low, then *L* is a link set that is well separated from the rest of the network. The escape probability of a link–node–link random walker is given by the following equations (Havemann, Gläser, & Heinz, 2019):

(1) $$P_{\mathrm{esc}}\left(L\right)\;=\frac{\sigma \left(L\right)}{k_{\mathrm{in}}\left(L\right)}$$

with

(2) $$\sigma \left(L\right)\;=\sum_{i=1}^{n}\frac{k_{i}^{\mathrm{in}}\left(L\right)k_{i}^{\mathrm{out}}\left(L\right)}{k_{i}}$$

and

(3) $$k_{\mathrm{in}}\left(L\right)\;=\sum_{i=1}^{n}k_{i}^{\mathrm{in}}\left(L\right)$$

$k_{i}^{\mathrm{in}}\left(L\right)$ and $k_{i}^{\mathrm{out}}\left(L\right)$ are the internal and external degrees of node *i* with respect to link set *L*. Their sum is the node’s total degree.

(4) $$k_{i}=k_{i}^{\mathrm{in}}\left(L\right)+k_{i}^{\mathrm{out}}\left(L\right).$$

Since our hypothesis network is small, and the disjoint clusters are already very suggestive, we were able to avoid the random components in the evolutionary approach of Havemann et al. (2017) and only made local searches in the cost landscape of *P_esc_* starting from the five disjoint clusters as seed link sets. Local searches go on the steepest path to the next local minimum in the cost landscape. In each step of a local search, we added this link to the set that resulted in the minimum cost. After reaching a local minimum, we continued the search, because cost landscapes are rough, and we did not want to get trapped in a local minimum that is only a few steps away from a deeper one. After expanding link sets, we excluded links until we found the final hypothesis clusters with the lowest escape probability. Further information on this approach is provided in Havemann et al. (2017).

## 3 RESULTS

**FIGURE 2:**
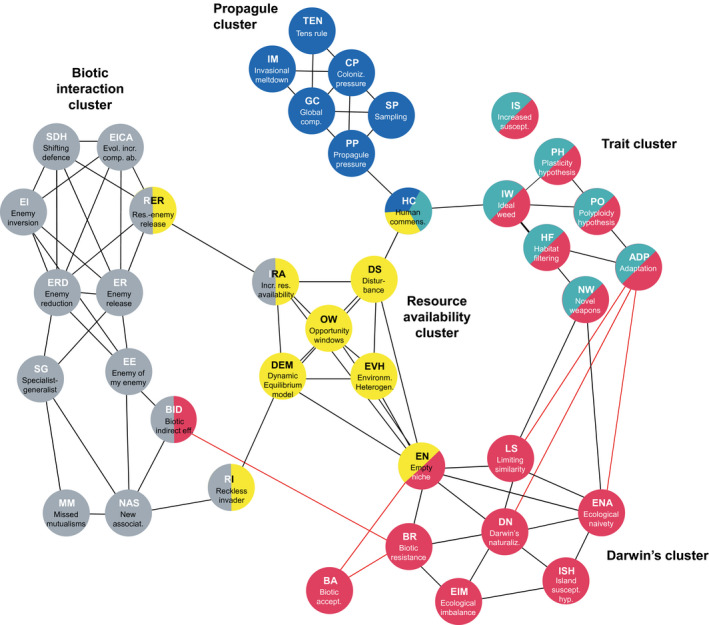
Network of 39 common hypotheses in invasion biology, clusters calculated with the local link‐clustering algorithm (hypothesis names are abbreviated as in Table 1 where details on each hypothesis are provided). Colours indicate membership of hypotheses to *concept clusters*. The representation is simplified in that, for example, the node empty niche (EN) appears to be split into two equal parts, while it actually belongs slightly more in Darwin’s cluster (6/11 = 55%) than in the resource availability cluster (5/11 = 45%); see Supporting Information Figure S2 for details. Similar hypotheses are connected with black lines, whereas contradictory hypotheses are connected with red lines [Colour figure can be viewed at wileyonlinelibrary.com]

The resulting consensus network included (a) five clusters covering 32 of the 39 hypotheses; (b) six *connecting hypotheses* acting as bridges between clusters (human commensalism, HC, connecting three clusters; and resource‐enemy release, RER, increased resource availability, IRA, reckless invader, RI, biotic indirect effects, BID, and empty niche, EN, each connecting two clusters); and (c) one hypothesis not connected with any other hypothesis in the network (increased susceptibility, IS, with the closest connection with the polyploidy hypothesis, PO; link = .48; Supporting Information Table S1) (Figure 2).

We named the five clusters the (a) ‘biotic interaction cluster’ accounting for nine full‐member hypotheses (i.e., without connecting hypotheses), (b) ‘Darwin’s cluster’ (seven full‐member hypotheses), (c) ‘trait cluster’ (six full‐member hypotheses), (d) ‘propagule cluster’ (six full‐member hypotheses) and (e) ‘resource availability cluster’ (four full‐member hypotheses) (Figure 2). The trait cluster is actually nested in Darwin’s cluster (cf. Supporting Information Appendix S1), hence one could also consider Darwin’s cluster to include 13 full‐member hypotheses that are further separated into two sub‐clusters. For simplicity, though, and because none of the other clusters includes sub‐clusters, we do not usually discriminate between first‐ and second‐level clusters here.

## 4 DISCUSSION

### 4.1 Hypothesis clusters

Each of the five clusters we identified encapsulates a main explanation why a non‐native species may become invasive (sensu Blackburn et al., 2011). The commonality among the hypotheses in the biotic interaction cluster is the role of interspecific (mostly negative) interactions in species invasion success. Most hypotheses in this cluster assume that natural enemies (i.e., predators, herbivores, parasites and pathogens) control species populations, so when a species is introduced to a new area, populations thrive because enemies are left behind. Similarly, Schulz, Lucardi, and Marsico (2019) recently offered a framework of hypotheses focusing on how enemies/antagonists affect invasion success. The lack of specific enemies in the recipient location gives an advantage to non‐native over native species (enemy release, ER) despite generalist enemies also reducing their performance. Some hypotheses in this cluster posit that enemy release allows non‐native individuals to reallocate resources from defences against natural enemies towards growth, fitness and competitive ability (evolution of increased competitive ability, EICA; shifting defence hypothesis, SDH). Mutualistic interactions with native species (e.g., pollinators, seed dispersers, mycorrhiza) also increase invasion success (Richardson, Allsopp, D'Antonio, Milton, & Rejmanek, 2000), whereas interspecific competition with the native species (reckless invader, RI) or a lack of mutualists (i.e., those missing compared to the invader’s home range) impede it (missed mutualism, MM).

The hypotheses in the resource availability cluster associate invasion success with invader access to resources, which is affected by abiotic and biotic conditions and their interaction (Catford et al., 2009 and references therein). The first three hypotheses (increased resource availability, IRA; disturbance, DS; opportunity windows, OW) centre on temporary increases in resource availability, which can result from a decline in resource uptake in the community and/or an increase in supply. Increased resource availability (IRA) and disturbance (DS) focus on fluctuations through time, whereas opportunity windows (OW) considers fluctuations in both space and time (see also Davis, Grime, & Thompson, 2000). High resource availability, even if only temporary, enables invader populations to become established, from which point they can continue to grow and spread. The dynamic equilibrium model (DEM) centres on interactions between disturbance and productivity, which collectively affect resource availability and strength of resource competition, and thus opportunities for invasion. While the underlying mechanism is arguably the same (sufficient resource availability), environmental heterogeneity (EVH) is phenomenological and pattern‐based, unlike the first four process‐based hypotheses. EVH essentially attributes invasion success to incomplete resource uptake by the resident community. This is because communities in ecosystems with high environmental heterogeneity are less likely to be saturated, such that associated resources remain unused (or under‐used). These available resources provide ripe opportunities for (effectively competition‐free) invasion by species having the appropriate niche. EVH is strongly linked with the empty niche hypothesis (EN; which follows Elton’s rather than Hutchinson’s niche concept, cf. Pulliam, 2000), a connecting concept between the resource availability cluster and Darwin’s cluster (Figure 2).

Many of the hypotheses in Darwin’s cluster have an eco‐evolutionary perspective on biological invasions, which highlights the importance of species’ evolutionary legacies in shaping the outcome of biotic interactions that result from species introductions. This is true for the ecological imbalance (EIM) hypothesis, which focuses on the evolutionary characteristics of both the region that receives the non‐native species and regions where that species is native. Another example is ecological naivety (ENA), which is also known as evolutionary naivety. Ecological niches are shaped evolutionarily, and many hypotheses in this cluster are related to species’ niches, either that of the non‐native species arriving in an ecosystem or that of the species assemblage composing the native community. Indeed, several of these hypotheses propose that non‐native species could only establish and potentially become invasive if they can occupy niches different from those of the native species, a theoretical concept developed by Shea and Chesson (2002). In practice, niche similarity or divergence has been characterized by species’ functional traits, given their link to resource acquisition, evolutionary fitness and ecosystem processes (Divíšek et al., 2018; Vidal‐Garcia & Keogh, 2017; Wang, Hu, Wang, Liu, & Yu, 2018), or by species relatedness, assuming that species niches are conserved in phylogenies (Prinzing, Durka, Klotz, & Brandl, 2001; Thuiller et al., 2010). In other words, the likelihood of a non‐native species becoming invasive is, according to these hypotheses, related to dissimilarities in the non‐native species’ characteristics with respect to the recipient community, and thus, associated with their resource use in the new environment. Furthermore, the relationship between species evolution, niche space and species traits explains why we found the trait cluster to be nested in Darwin’s cluster.

The trait cluster includes six hypotheses that focus on traits to explain why a non‐native species may become invasive. This is a topic of long‐standing interest within invasion biology, from its very onset, as it is thought that certain species traits are associated with invasiveness (Baker, 1974; Capellini, Baker, Allen, Street, & Venditti, 2015; Mahoney et al., 2015; Pyšek & Richardson, 2007; van Kleunen, Weber, & Fischer, 2010). The hypotheses included within this cluster consider traits that can help non‐native species to generally become invasive (ideal weed, IW), to compete with native species (novel weapons, NW), or to adapt to the novel conditions found in their introduced ranges (adaptation, ADP; polyploidy hypothesis, PO; plasticity hypothesis, PH; habitat filtering, HF).

Finally, the hypotheses in the propagule cluster relate the numbers of introduced non‐native species or individuals to the probability that they will become invasive. The propagule pressure hypothesis (PP) operates at the population level and suggests that the likelihood of a non‐native population being able to establish increases with the number of individuals of that species being introduced. Several potential mechanisms underpin the propagule pressure hypothesis, all of which invoke the ability of larger numbers of individuals to overcome random, stochastic forces to ensure population persistence. The other five hypotheses operate at the community level and suggest that greater numbers of species become invasive if greater numbers of species are introduced (colonization pressure, CP; global competition, GC; invasional meltdown, IM; sampling, SP; tens rule, TEN). Similar to the propagule pressure hypothesis, these hypotheses assume that the chance of some species experiencing favourable ecological conditions increases with greater numbers of species introductions.

In a nutshell, each hypothesis cluster focuses on a particular perspective on biological invasions. A related question is if research done within each cluster focused on particular taxonomic groups or habitats. Four of the 10 hypotheses for which Jeschke and Heger (2018: tables 17.2, 17.3; underlying data available at http://www.hi‐knowledge.org) gathered data are in Darwin’s cluster (Darwin’s naturalization, DN; limiting similarity, LS; biotic resistance, BR; island susceptibility hypothesis, ISH; the remaining three are in each of the other clusters) and three are in the propagule cluster (propagule pressure, PP; tens rule, TEN; invasional meltdown, IM), which allows us to make a first assessment of potential research biases for these two clusters. Indeed, relatively more studies in Darwin’s cluster focused on plants (67.3%, based on percentage values for each hypothesis) than in the propagule cluster (37.7%). Similarly, while only an average of 2.6 and 8.4% of studies addressing hypotheses in Darwin’s cluster focused on marine and freshwater habitats, respectively, studies in the propagule cluster are less biased; here, an average of 10.2 and 18.1% of the studies focused on marine and freshwater habitats, respectively. Thus, even these limited data show strong differences in research focus between hypothesis clusters. These differences indicate biases and different types of knowledge gaps in each particular cluster. More detailed explorations of these biases and gaps are certainly warranted.

### 4.2 Connecting hypotheses

While clusters of hypotheses can reflect fertile areas of similar research questions, *connecting hypotheses* are nodes that apparently overlap with, or logically connect, two or more clusters. Thus, these nodes offer logical links between major areas of research within the field. For example, the increased resource availability (IRA) hypothesis connects the resource availability and biotic interaction clusters. The former cluster is concerned with changing conditions and opportunities, such as shifts in resource uptake and supply, whereas the latter cluster emphasizes the importance of a favourable biotic context in which enemies no longer constrain the population growth of the invader. In particular, non‐native species must often co‐opt limiting resources from native competitors in order to maintain population persistence; thus, the IRA hypothesis is linked to the biotic interaction cluster. Similarly, the human commensalism hypothesis (HC) logically connects to the trait cluster by recognizing the importance of trait plasticity and pre‐adaptation for surviving human‐mediated disturbances and land use (e.g., agriculture), and for exploiting human transportation systems. Human commensalism also implies greater opportunities for propagule dispersal, hence the link to the propagule cluster. Finally, human commensalism reflects the ability of successful invaders to opportunistically exploit human‐mediated disturbance events – which promote enemy release and resource release via the loss of resident predators and competitors.

To our knowledge, such connecting hypotheses have not been identified before. Thus, in addition to the largest hypothesis network created to date using a state‐of‐the‐art method, another novel result of this study is the identification of hypotheses that connect hypothesis clusters, and thus, serve as conceptual bridges in invasion biology. Such conceptual bridges play a key role in advancing scientific goals, and an important extension of the work presented here will be to identify them also at the level of disciplines (in addition to the level of hypotheses shown here). For example, Jeschke (2014) outlined that the ‘diversity‐stability hypothesis which states that ecosystems with high biodiversity are more stable than ecosystems with low biodiversity’ (p. 1,230) is a more general formulation of the biotic resistance hypothesis (BR) in invasion biology where ‘stability’ is resistance against non‐native species. The same idea can be found in disease ecology in which ‘stability’ is resistance against pathogens (Lively, 2010; Sommer, 2005). Identifying more such connecting hypotheses will help us to better bridge research within and between research fields.

### 4.3 Comparison with previous hypothesis networks in invasion biology

Some papers previously categorized hypotheses and concepts in invasion biology (e.g., Catford et al., 2009; Chabrerie et al., 2019; Gurevitch, Fox, Wardle, Inderjit, & Taub, 2011; Schulz et al., 2019) or visualized them in the form of networks (Enders et al., 2018, 2019; Enders & Jeschke, 2018). Although useful for providing a first overview, these previous approaches to creating hypothesis networks in invasion biology had several limitations that were overcome by our consensus approach. In particular, here we assembled a fairly large and diverse group of experts constructing the consensus network who were offered the opportunity to discuss why they consider hypotheses to be logically linked, and they could differentiate between positive linkages, negative linkages or unlinked hypotheses. Another advantage to our approach here is that, unlike quantitative bibliometric approaches, it does not depend on a large literature database. Finally, our consensus approach reflects how the concepts are being currently used in practice, and presently perceived and interpreted by experts working in the field. This is in contrast to the bibliometric approach, which is based on historic citation patterns.

The networks resulting from the consensus approach used in this paper and those from previous bibliometric approaches (see Enders et al., 2019) are quite similar; however, due to the outlined benefits of the consensus approach, we recommend this approach to create networks of hypotheses and concepts in a research field.

## 5 CONCLUSIONS AND OUTLOOK

Our hypothesis network visualizes the conceptual structure of invasion biology. It displays relationships among invasion hypotheses that can in turn be tested with empirical studies. A next step should be to offer both the network and empirical studies as interactive tools online. This output provides the opportunity to (a) bridge the gap between theoretical‐conceptual and empirical work, and (b) offer a visual and user‐friendly interface to explore the knowledge depth, gaps and redundancies of the field. In this way, it would be immediately visible which hypotheses are empirically supported under which circumstances, particularly when dividing the 39 hypotheses into more specific sub‐hypotheses following the hierarchy‐of‐hypotheses approach (Jeschke & Heger, 2018). A first step in this direction is available at the website http:// www.hi‐knowledge.org. Another helpful extension of the network will be to visualize taxonomic and habitat biases among the clusters and hypotheses.

The clusters in our network provide a clear, simplified summary of the main mechanisms that, according to current theory, govern the introduction, establishment and spread of invasive species. The clustering highlights that the field is currently dominated by attention to antagonistic interactions between non‐native and native species; it recognizes the stochastic nature of invasions through spatio‐temporal variation in biotic and abiotic conditions (resource availability cluster), as well as in propagule supply and filtering (propagule cluster); and part of the foundation of the field is built upon venerable hypotheses arising from Darwin and Elton (cf. Table 1). This method could also be used to identify temporal trends in the concepts, that is, when hypotheses were proposed and coined, and when they experienced most empirical examination. One might see moving waves of research effort through the network as research fashions and techniques change.

Further, a hypothesis network such as the one constructed here can guide a researcher working on one hypothesis to explore potentially relevant ideas and literature concerning hypotheses that are nearby in the cluster, and to highlight important covariables that should be used in analyses that might otherwise be overlooked. The researcher will also be pointed towards critical research and knowledge gaps. Similarly, a hypothesis network avoids the formulation of additional repetitive hypotheses. Anyone who wants to propose a new hypothesis or mechanism to the field can consult the network to see where the new contribution would be located and if it overlaps with existing ones.

Our hypothesis network is not definitive. It does not include all existing invasion hypotheses (cf. Chabrerie et al., 2019) and obviously cannot include hypotheses that do not yet exist. Invasion biology will further develop, and so will its conceptual structure. For example, the importance of humans as drivers of invasions does not play a dominant role in the current network. Humans clearly play a key role in the propagule cluster, as it highlights the importance of non‐native species’ introductions which are mediated by humans. We expect that in the future and with increasing research efforts focusing on the Anthropocene, the field will focus more on the role of humans (Kueffer, 2017) and how we can better integrate related, but still largely isolated research fields (Heger et al., 2019; Vaz et al., 2017). For the latter, the connecting hypotheses highlighted in this study will play an important role. Since the conceptual map presented here is a stepping stone towards the future of the field, it should be regularly revised and extended.

Finally, the consensus approach outlined here can be applied to any research field. We strongly encourage its application particularly in disciplines where, as in invasion biology, there are so many hypotheses and concepts that it is hard to gain an overview without a navigation tool like a hypothesis network. Connecting an increasing number of hypothesis networks could facilitate cross‐disciplinary research by revealing overlaps and joint ideas, enhancing the understanding of basic ideas and transfer of knowledge. A resulting growing atlas of knowledge could thus help address complex problems like multi‐causality in biodiversity change (Sala et al., 2000; Settele et al., 2005), and to build a solid basis for tackling the current environmental crisis. Such an atlas would also reveal hypotheses and concepts that connect disciplines, helping researchers to find out if colleagues from another discipline have already come up with concepts and ideas to potentially solve challenges in their own field. Therefore, we call on researchers across scientific disciplines to create conceptual maps for their fields. Let’s then connect these maps to jointly build an atlas of knowledge.

## BIOSKETCH

**Martin Enders** is a young biologist and philosopher who focuses on invasion biology. He is especially interested in the synthesis and visualization of different hypotheses and concepts to construct ‘maps’ of research fields. His broad research interests include theoretical ecology, biological invasions and philosophical biology.

## Supporting information


TABLE S1

Appendix S1

## Data Availability

The data created in this study and underlying the presented analyses are freely available online in the Supporting Information accompanying this publication.
