# Supplementary material for: A conceptual map of invasion biology: Integrating hypotheses into a consensus network
Source: Glob Ecol Biogeogr. 2020 Mar 25;29(6):978–91. doi: 10.1111/geb.13082 (PMC8647925; doi:10.1111/geb.13082)
Supplement: Supplementary file 2 — Appendix S1 [file GEB-29-978-s002.pdf]

Supporting Information Appendix S1 to Enders et al.: A conceptual map of invasion biology: Integrating hypotheses into a consensus network

Classical clustering approach: node clustering

A classical clustering approach for network analysis is node clustering where nodes are clustered rather than their links (the alternative link-clustering approach that we used for Figure 2 clusters links rather than nodes). We tested the utility of node clustering for our hypothesis network, applying four algorithms commonly used to detect network structures: (i) the Girvan and Newman (2002) algorithm (igraph function 'cluster_edge_betweenness'); (ii) an algorithm proposed by Clauset et al. (2004) (igraph function 'cluster_fast_greedy'); (iii) the "Walktrap" algorithm suggested by Pons and Latapy (2005) (igraph function 'cluster_walktrap'); and (iv) an algorithm suggested by Newmann (2006) (igraph function 'cluster_leading_eigen').

The clusters returned by these four established algorithms were inconsistent (Fig. S1). These inconsistencies were largely due the fact that some hypotheses did not seem to be part of any single cluster but were instead bridging clusters. For example, the human commensalism hypothesis (HC) seems to connect three clusters, but each algorithm assigned it to a different single cluster, as these ordinary algorithms are unable to split nodes into several clusters.

One possible solution to overcome these inconsistencies is to inspect the results of these different algorithms and combine them manually by identifying (a) stable clusters, i.e. those groups of nodes that are assigned to the same cluster by all algorithms, and (b) nodes that are assigned to different clusters (*connecting concepts*). However, link clustering has become available as an alternative and more elegant approach for identifying stable clusters and connecting concepts, so we used it in this study.

References

Clauset, A., Newman, M. E. J., & Moore, C. (2004). Finding community structure in very large networks. *Physical Review E, 70*, 066111. doi:10.1103/PhysRevE.70.066111

Girvan, M., & Newman, M. E. J. (2002). Community structure in social and biological networks. *Proceedings of the National Academy of Sciences of the United States of America, 99*, 7821-7826. doi:10.1073/pnas.122653799

Newman, M. E. J. (2006). Modularity and community structure in networks. *Proceedings of the National Academy of Sciences of the United States of America, 103*, 8577-8582. doi:10.1073/pnas.0601602103

Pons, P., & Latapy, M. (2005). Computing communities in large networks using random walks. *Computer and Information Sciences - Iscis 2005, Proceedings, 3733*, 284-293.

Additional supplementary figures

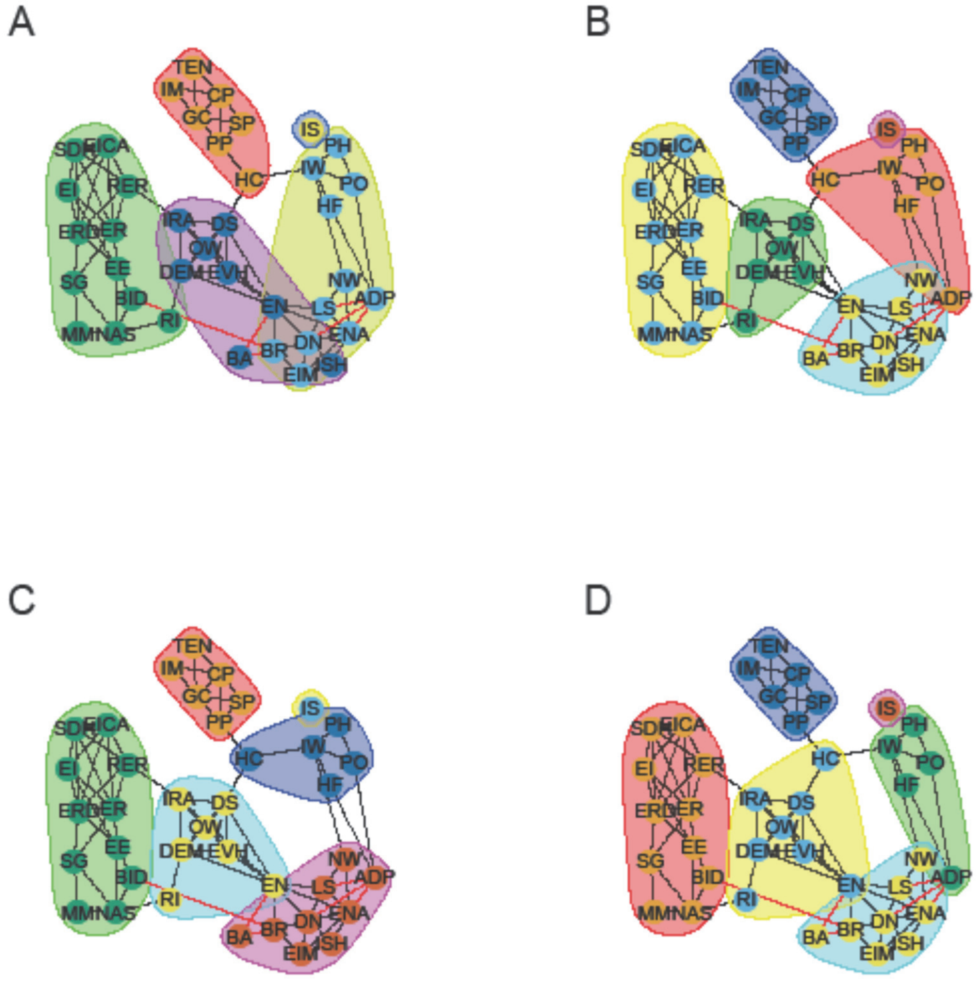

Figure S1. Results of four established algorithms to find node clusters in the network of 39 invasion hypotheses. The algorithms achieved similar levels of modularity: (A) 0.599 (edge betweenness), (B) 0.611 (fast greedy), (C) 0.613 (leading eigen), (D) 0.612 (Walktrap). Similar hypotheses are connected with grey lines, whereas contradictory hypotheses are connected with red lines. See Table 1 in the main article for a description of the hypotheses.

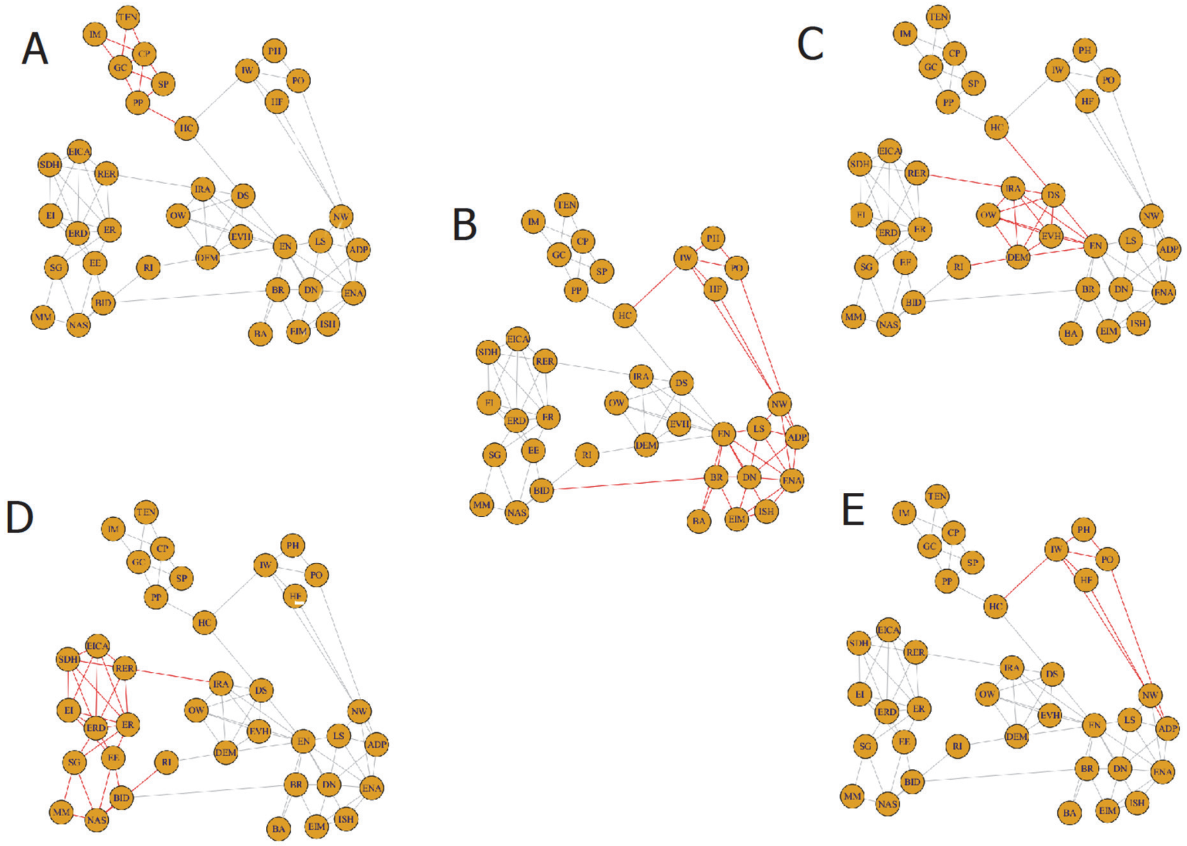

Figure S2. Link clusters of the local link-clustering algorithm. Red lines connect the hypotheses in the following clusters: (A) Propagule cluster, (B) Darwin's cluster and Trait cluster, (C) Resource availability cluster, (D) Biotic interaction cluster and (E) Trait cluster.
